# Preparation and Characterization of Electrospun Polylactic Acid (PLA) Fiber Loaded with Birch Bark Triterpene Extract for Wound Dressing

**DOI:** 10.1208/s12249-021-02081-z

**Published:** 2021-07-20

**Authors:** Tianyuan Fan, Rolf Daniels

**Affiliations:** 1 The State Key Laboratory of Natural and Biomimetic Drugs, School of Pharmaceutical Sciences, Peking University, Beijing, China; 2 Beijing Key Laboratory of Molecular Pharmaceutics and New Drug Delivery Systems, School of Pharmaceutical Sciences, Peking University, Beijing, China; 3 Department of Pharmaceutical Technology, University of Tübingen, Auf der Morgenstelle 8, D-72076 Tübingen, Germany

**Keywords:** electrospinning;, polylactic acid;, triterpene extract;, wound dressing;, drug release

## Abstract

Drug-loaded electrospun fibers have attracted increasing attention as a promising wound dressing material due to their capability of preventing from infections and inflammation and maintaining an appropriate environment for wound healing. In this study, polylactic acid (PLA), which is widely used in wound management, was chosen as electrospinnable polymer. A triterpene extract (TE) from the outer bark of birch known for its anti-inflammatory, antiviral, antibacterial, and wound healing effects was chosen to produce TE-loaded PLA electrospun fibers for wound dressing. A binary solvent system of dichloromethane (DCM) and dimethyl sulfoxide (DMSO) was employed, and the ratio of the solvents was optimized for preparing smooth and uniform fibers. The morphology of TE-loaded PLA electrospun fibers was investigated by scanning electron microscopy (SEM). The entrapment of TE in PLA fibers was confirmed by confocal laser scanning microscopy (CLSM). Differential scanning calorimetry (DSC) and X-ray diffraction (XRD) were used to analyze the solid state of TE in PLA fibers. The release behavior of TE was assayed by a shaking flask method for a period of 96 h. The results revealed that TE-loaded electrospun PLA microfibers could be reliably prepared and are promising future candidates in wound therapy.

## INTRODUCTION

Electrospinning technology has attracted much attention for wound healing in recent years due to its simplicity, reproducibility, and cost-efficacy (1). In a typical electrospinning process, a high-voltage electric field is applied between the polymeric solution and a metallic collector which act as a counter electrode. Due to electrostatic attraction, a nano- or microfiber is formed through liquid jetting. The solvents from the polymer solution evaporate during the travel in the electrical field and a solid fiber reaches the collector (1). Electrospun fiber-based dressings not only maintain wound hydration, absorb excess wound exudate, minimize wound trauma, and barrier external microorganisms in traditional way, but also mimic the native dermal extracellular matrix (ECM) and have high surface area-to-volume ratio as well as high porosity, which tends to create positive environment to reduce infection and inflammation and to promote wound healing (2). Besides, electrospun fibrous membranes can act as drug delivery systems by loading therapeutic agents including antimicrobial, anti-inflammatory, and antioxidant species to prevent skin infections and improve wound healing process (3,4).

Polylactic acid (PLA) is so far the most extensively researched and utilized biodegradable aliphatic polyester in human history. It plays an increasingly important role in medical applications owing to its unique properties comprising biodegradability, biocompatibility, and complete reabsorbability. PLA has been used in a wide range of applications related to wound management, such as surgical sutures, healing dental extraction wounds, and preventing postoperative adhesions (5,6). PLA appears as L-PLA (PLLA), D-PLA (PDLA), racemic mixture D, L-PLA (PDLLA), and stereocomplex PLA (sc-PLA) (7,8). All the four kinds of PLA can be used to prepare electrospun fibers (9). Electrospun PLA fibers have been reported to have positive effect on collagen deposition, angiogenesis, and inflammation, and accelerate the rate of wound closure compared to commercial gauzes (10). In another report, electrospun PLA scaffolds with minced skin grafts together have been used as skin substitute for surgical repair of skin defects in plastic and reconstructive surgery. The skin cells were confirmed to migrate along the PLA fibers of the scaffold and new collagen was formed (11). Drug-loaded electrospun PLA fibers have also been reported to enhance wound healing in literature. Cyclosporine A-loaded electrospun PLA nanofibers effectively suppressed inflammation and neovascularization compared with drug-free PLA nanofibers in the treatment of alkali-injured rabbit cornea (12). Aligned porous electrospun PLA fibrous membranes containing dimethyloxalylglycine-loaded mesoporous silica nanoparticles were demonstrated to simulate the healing of diabetic wound *in vitro* (13). Nitrofurazone-loaded electrospun PLA/sericin-based dual-layer fiber mats performed better in decreasing wound size in rats than commercial non-woven dressing did (14). An electrospun PLA scaffold integrating nucleic acid delivery produced significant enhancements in wound healing in mice (15).

Triterpene extract (TE) from the outer bark of birch consisting of betulin, betulinic acid, lupeol, erythrodiol, caffeoyl-betulin, and oleanolic acid shows anti-inflammatory, antiviral, antimicrobial, and other pharmacological activities (16–18). TE and betulin were reported to address the inflammatory phase of wound healing by transient upregulation of several pro-inflammatory mediators and enhance the migration of keratinocytes in the second phase of wound healing (19). TE-based oleogel (Episalvan) can affect all the three phases of wound healing, including inflammation, migration, and differentiation, and promote wound healing in several clinical phase II and phase III studies. It was approved by the European Medicine Agency (EMA) for the European Economic Area in 2016 to accelerate wound closure in partial thickness wounds, which opened up the area of application of wound healing for phytotherapy in plastic surgery and especially in burn medicine (18,20).

Combining the advantages of electrospinning fibers and the pharmacological activities of TE for wound healing, TE-loaded PLA electrospun fibers were fabricated for wound dressing in this study. The electrospun fibers were then characterized *in vitro* by scanning electron microscopy (SEM), confocal laser scanning microscopy (CLSM), differential scanning calorimetry (DSC), and X-ray diffraction (XRD). The drug release behavior was also investigated. The methods used and results obtained in this study were discussed carefully by searching and comparing with a number of related literatures.

## MATERIALS AND METHODS

### Materials

Polylactic acid (PLA L207S) was purchased from Evonik, Germany. A pharmaceutical grade birch bark triterpene extract from the outer bark of birch (TE) was obtained from Amryt AG, Niefern-Öschelbronn, Germany, comprising betulin 81.60%, lupeol 2.08%, betulinic acid 3.84%, erythrodiol 1.05%, oleanolic acid 0.97%, betulinic acid methyl ester 0.52%, and undisclosed substances 9.94%. Betulin was supplied by Sigma-Aldrich. Dimethyl sulfoxide (DMSO, laboratory reagent grade), dichloromethane (DCM, HPLC grade), and acetonitrile (HPLC gradient grade) were purchased from Fisher Scientific, UK.

### Methods

#### Preparation of Polymer Solution

To prepare polymer solution for electrospun TE-free fibers, PLA was dissolved in a mixture of DCM and DMSO (4:1, v/v) with magnetic stirring at room temperature. The final concentration of PLA solution was 70 mg/mL.

To prepare a polymer solution for electrospun TE-loaded fibers, PLA and TE were separately dissolved in a mixture of DCM and DMSO (4:1, v/v) with magnetic stirring at room temperature. The solutions of PLA and TE were then mixed homogenously. The final concentration of PLA and TE in the mixed solution was 64 mg/mL and 4 mg/mL, respectively.

#### Electrospinning Process

A basic series electrospinning unit (Nanolab, Malaysia) was employed to prepare electrospun fibers. The polymer solution was placed in a 10-mL plastic syringe (Luer Lock Solo syringe, B Braun, Germany) pumped at a speed of 0.7 mL/h for electrospinning of TE-loaded fibers or 1.2 mL/h for electrospinning of TE-free fibers. The inner diameter of a blunt needle fixed on the syringe was 0.8 mm. A rotating drum collector was located 10 cm from the needle tip, covered with aluminum foil for deposition of fibers. Its rotation speed was adjusted to 1000 rpm. The voltage between the needle and the collector was set at 9 kV for TE-loaded fibers or 11 kV for TE-free fibers. The electrospinning experiments were performed at room temperature.

#### Scanning Electron Microscopy

The morphology of the electrospun fibers was analyzed by scanning electron microscopy (SEM) Zeiss DSM 940 A equipped with a Frame Grabber (Orion 5.25). The collected electrospun fiber films were cut into pieces (about 0.7 cm × 0.7 cm) and applied to a double-sided, conductive, and adhesive tape. The samples were covered with gold in a sputter coater (Bio Rad E5100) at 2.1 kV and 20 mA (4 × 60 s). The acceleration voltage was 5 kV.

To determine the average diameter as well as the diameter distribution of the optimal TE-free and TE-loaded electrospun fibers, 100 fibers were selected randomly from the SEM images with a magnification of 5000× and measured using image analysis software Image J (21).

#### Confocal Laser Scanning Microscopy

The fluorescence of TE was measured by fluorescence spectrophotometer in our preliminary experiment. The excitation wavelength and emission wavelength of TE were presented at 345 nm and 426 nm, respectively. Subsequently, confocal laser scanning microscopy (CLSM) (ZEISS LSM 880 with Airyscan, Germany) was employed to visualize the distribution of fluorescence originating from TE in the TE-loaded fibers, and TE-free fibers were used as contrast. TE-free fibers and TE-loaded fibers were separately fixed on microscope glass slides and examined directly at an excitation wavelength of 405 nm. Images in XY plane obtained from the surface of fibers were recorded and processed by the aid of ZEN 2.3 software.

#### Differential Scanning Calorimetry

Differential scanning calorimetry (DSC) analysis was performed by a DSC 820 calorimeter (Mettler-Toledo GmbH, Giessen, Germany). Samples of TE, PLA, TE-free fibers, and TE-loaded fibers were separately placed in 40-μL aluminum crucibles (Mettler-Toledo GmbH, Switzerland); an empty crucible was used as reference. The instrument was calibrated with indium from −94 to 490°C. An 80 mL/min nitrogen purging gas flow was used. Typically, after equilibration for 3 min at −10°C, the temperature was raised to 270°C, and then decreased to −10°C and raised to 270°C once more at a rate of 20 K/min.

#### X-ray Diffraction

X-ray diffraction (XRD) measurements of TE, PLA, TE-free fibers, and TE-loaded fibers were performed in a diffractometer (MiniFlex 600, Rigaku, Japan). The generator was operated at voltage of 40 kV and current of 15 mA. The spectrum was collected at a diffraction angle 2θ ranging from 0° to 90° with the step size 0.02° at the rate of 6°/min.

#### Dissolution Tests

About 2 mg of the TE-loaded fibers was weighted precisely and put in a plastic centrifugal tube. Subsequently, 10 mL dissolution medium were added. As TE is not sufficiently soluble in water and in order to maintain sink conditions, pH 7.4 phosphate buffered ethanol 60% (v/v) was used as dissolution medium (22). The tube was then shaken in an incubator (INFORS HT, Minitron, Switzerland) at a speed of 50 rpm at 32 ±1°C. One milliliter of the released medium was withdrawn at 0.5, 1, 2, 4, 6, 8, 24, 48, and 96 h, respectively, and replaced by fresh medium at the same volume and temperature. Finally, the fibers in medium were sonicated for 1.5 h to completely release TE from the samples. The dissolution tests were repeated in triplicate for the TE-loaded fibers.

High performance liquid chromatography (HPLC) (Shimadzu, Kyoto, Japan) method was used to measure the concentration of betulin (the main component of TE, which acts as an analytical marker) in the dissolution medium according to the previous report (23). Chromatographic separation was achieved on Nucleosil^®^ C18 RP column (125 mm × 4 mm, Macherey-Nagel, Dueren, Germany) kept at 40°C. The mobile phase was composed of solvent A (0.1% phosphoric acid in water) and solvent B (acetonitrile). A gradient elution program was as follows: 0–13 min, 75% B; 14–18 min, a linear increase from 75 to 90% B; 19–35 min, 90% B; 36–40 min, a linear decrease from 90 to 75% B; and 41–45 min, 75% B. The flow rate was 1.2 mL/min, the volume of injection was 100 μL and the UV detection wavelength was 210 nm. A calibration curve of the peak area and known concentration of betulin was obtained (A = 45056 C + 8258.8, R^2^ = 0.9990).

## RESULTS AND DISCUSSION

### Preparation of Electrospun Fibers

To fabricate eletrospun fibers, PLA can be found to dissolve in single (24–26), binary (27–29), or ternary (12) solvent in literatures. Chloroform and DCM are the most frequently used solvents. They can be used alone (24,25) or mixed with other solvents, such as ethanol (27), tetrahydrofuran (28), and dimethylformamide (29). In this study, the raw PLA can be dissolved in chloroform, DCM, or hexafluoroisopropanol (HFIP). DCM was selected to prepare PLA solution considering it has lower toxicity than chloroform and it is more frequently used than HFIP. DMSO, an organic solvent with lower saturated vapor pressure and high boiling point, was added to DCM to prevent the blockage of spinneret needle caused by the quick evaporation of DCM when it was used alone. A similar phenomenon and method can also be found in preparing electrospun poly(butylene succinate) fibers (30).

**Fig. 1:**
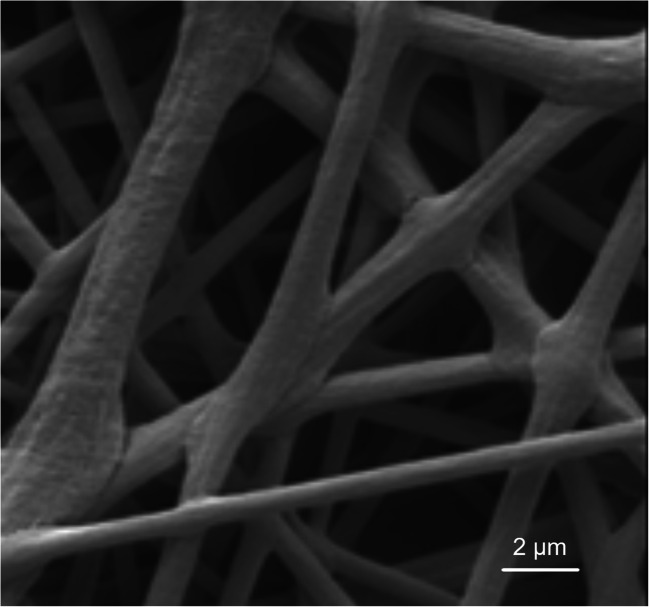
SEM image of TE-free PLA electrospun fibers prepared by using mixed solvents of DCM and DMSO at ratio of 16:9 (v/v)

The ratio of DCM and DMSO was investigated in our preliminary experiment. When the mixture of DCM and DMSO at ratio of 16:9 (v/v) was used to dissolve PLA, the electrospun TE-free PLA fibers presented splice-like welding from the SEM image (Fig. 1). The morphology of TE-free PLA fibers was greatly improved with the ratio of DCM and DMSO changed to 4:1 (v/v). The TE-loaded PLA fibers were then prepared by keeping the same ratio of the mixed solvents and slightly adjusting the voltage applied.

### Morphology and Diameter

**Fig. 2:**
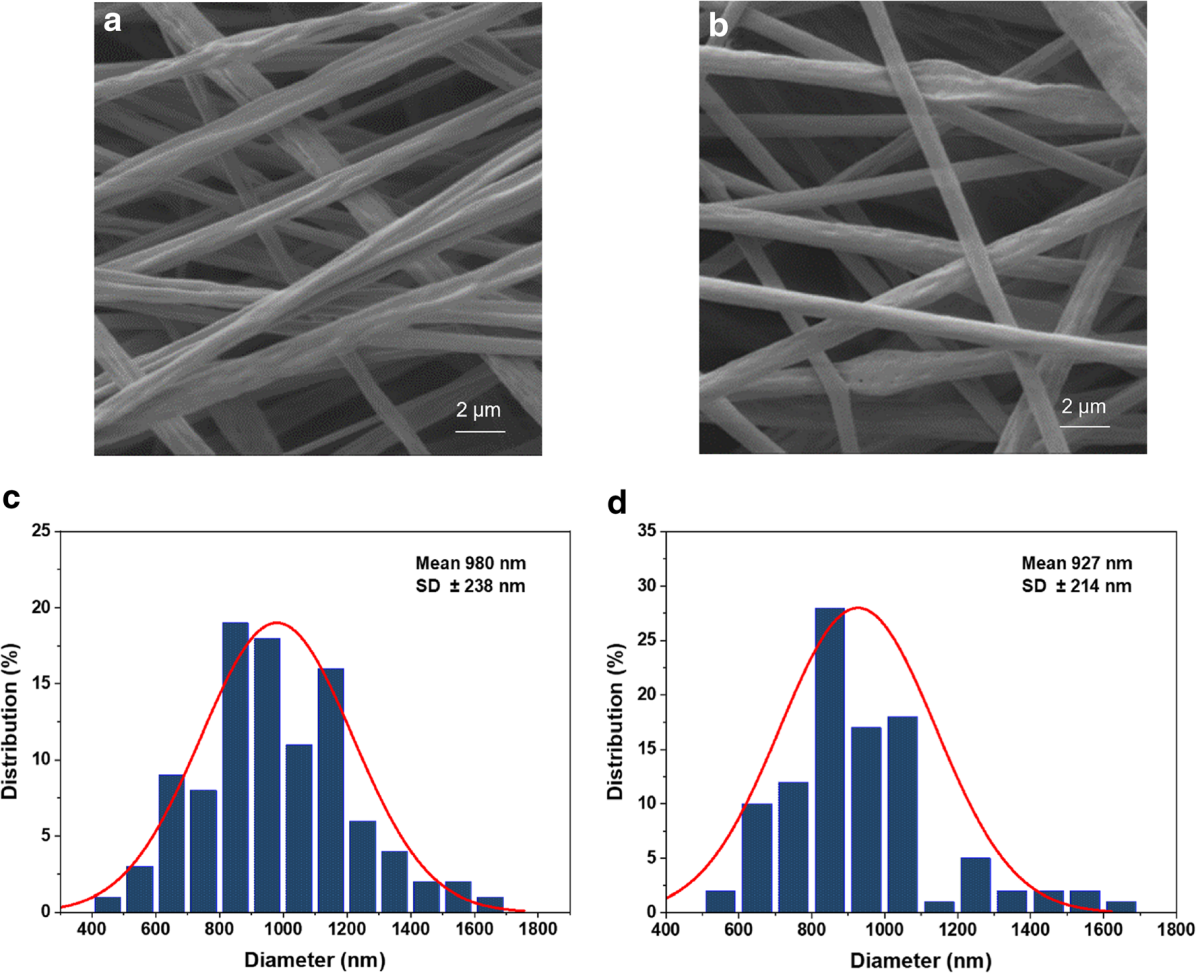
SEM images and diameter distribution of TE-free (**a**, **c**) and TE-loaded (**b**, **d**) PLA electrospun fibers

The SEM images of optimal TE-free and TE-loaded electrospun fibers are given in Fig. 2. Both of the fibers are randomly oriented. The TE-free fibers are more homogenous than TE-loaded fibers in which small amount of bead-like structure tends to occur. No signs are found that TE is deposited on the surface of TE-loaded fibers, indicating that TE is successfully entrapped within the matrix of PLA.

SEM images revealed that the diameters of TE-free and TE-loaded fibers are within the range from 411 to 1615 nm. The average diameters of TE-free fibers is 980±238 nm and are in good accordance with published data where a diameter of PLA electrospun fibers of 894.6±292.3 nm was reported when a 6 % PLA solution was used (10). The average diameter of TE-loaded fibers (927±214 nm) was found to be slightly but not significantly smaller when compared to TE-free fibers. The diameter of electrospun fibers can be found to be decreased, increased, or unchanged after drug loading in literatures. For example, the entrapment of clarithromycin (at 5 wt%) decreased the diameter of blank PLA fibers from 1091.8±223.0 nm to 408.4±112.8 nm (31). The loading of dexamethasone (at 3.5 wt%) on electrospun fibers of PLA increased the diameter from 2.13±0.3 μm to 2.91±0.5 μm (32). The addition of cyclosporine A (10 wt%) did not influence the diameter of electrospun PLA fibers which could be kept in the range from 290 to 539 nm (33). The change of diameter is supposed to depend on the impact of drug to the polymer solution composites in the aspects of conductivity, viscosity, and surface tension (31). The average diameters of TE-free and TE-loaded fibers in this study are both within the diameter range of electrospun PLA fibers mentioned above.

### Confocal Laser Scanning Microscopy

**Fig. 3:**
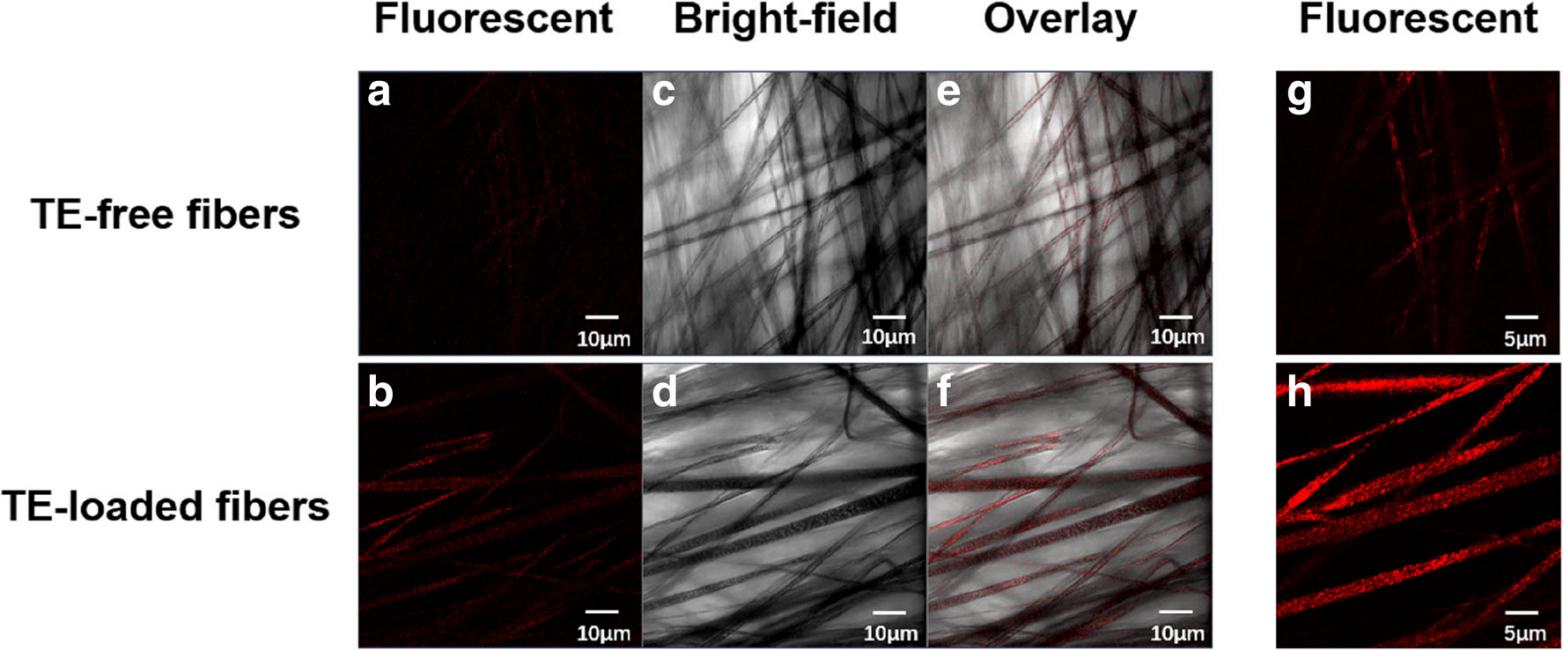
Examples of typical CLSM images of TE-free and TE-loaded PLA electrospun fibers at two magnifications: × 100 (**a**-**f**) and ×180 (**g**-**h**)

Examples of typical CLSM images of TE-free and TE-loaded electrospun fibers are shown in Fig. 3, where the fluorescent, bright-field, and merged images can be seen. The fluorescence intensity presented in TE-loaded fibers is much higher than that in TE-free fibers, especially in the fluorescent images at higher magnifications (180×). As the fluorescence detected represents TE, the result of CLSM confirms that TE was successfully loaded in the electrospun fibers of PLA.

CLSM has been used to examine the core-shell morphology of electrospun fibers occasionally (34,35). Fluorescent agents were first added to polymer solution of the core and/or shell, and the location of fluorescence in the electrospun fibers was then observed by CLSM. Fluorescence microscope has also been employed to observe the loading of moxifloxacin (self-fluorescent) on Eudragit L100 electrospun fibers (36). In this study, CLSM is applied to prove the entrapment of TE in PLA electrospun fibers and no additional fluorescent agent is added because TE has fluorescent property itself.

### Differential Scanning Calorimetry

**Fig. 4:**
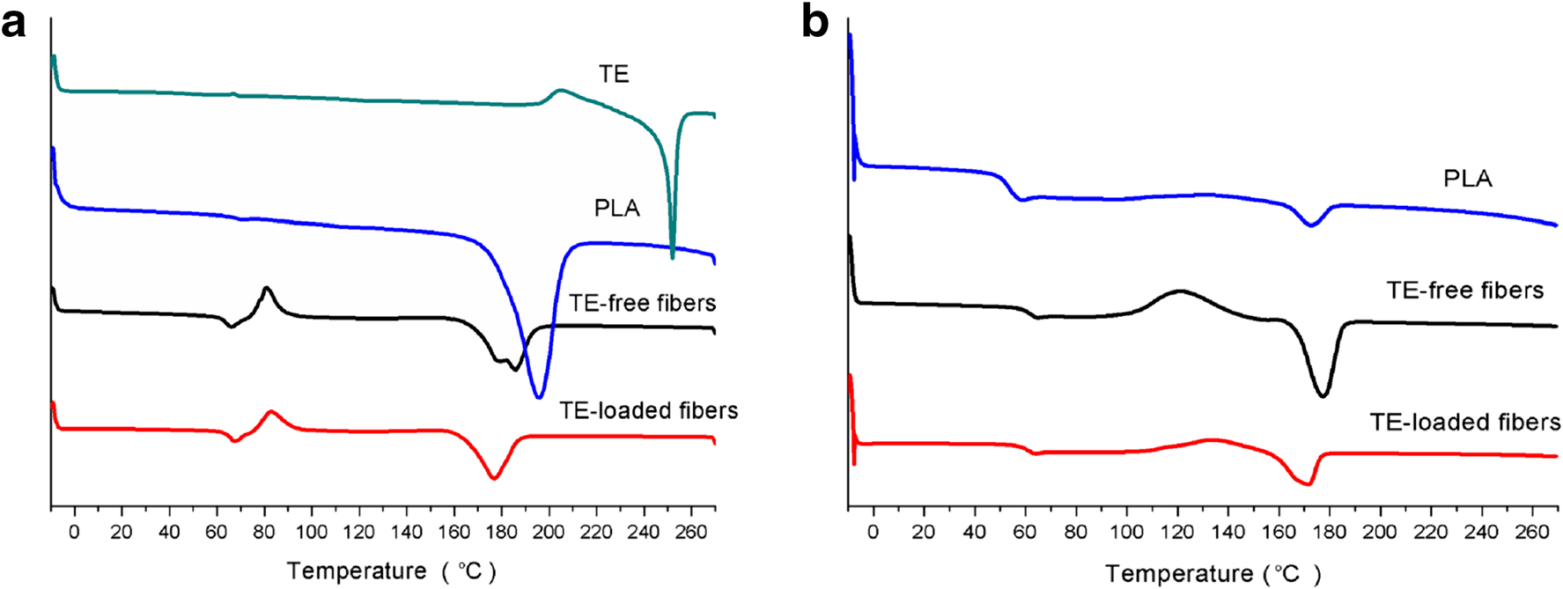
DSC pattern of TE, PLA, TE-free, and TE-loaded PLA electrospun fibers in the first (**a**) and PLA, TE-free, and TE-loaded PLA electrospun fibers in the second (**b**) heating process.

A melting peak of TE is detected at approximately 252°C during the heating process (curve is shown in Fig. 4), which is in accordance with that of betulin in previous report (37,38). The result is reasonable because the main component of TE is betulin (18,20). As the peak of TE does not appear in the DSC thermograms of TE-loaded PLA fibers (Fig. 4), TE is supposed to disperse in PLA fibers as amorphous solid (39) or in a molecularly dispersed state.

To eliminate the heating history of polymer, PLA, TE-free, and TE-loaded fibers underwent two heating processes (40). Fig. 4 shows the DSC pattern of PLA, TE-free, and TE-loaded fibers for the first and second heating process, respectively, and the corresponding glass transition temperature (T_g_), cold crystallization temperature (T_c_), and melting point (T_m_) are displayed in Table 1. In the second heating scan, the DSC measurements reveals two endothermic peaks of PLA at 52.2°C and 173.0°C corresponding to T_g_ and T_m_, respectively. The values are within the range of T_g_ (40–70°C) and T_m_ (130–230°C or typical 170–180°C) of PLA reported in literature (5,41). Moreover, the T_g_ is close to that of PLA Resomer 207 (same with PLA used in this study) in a previous report (42). As for TE-free and TE-loaded electrospun PLA fibers, the T_g_ are at 60.6°C and 60.1°C, and the T_m_ are at 177.3°C and 171.3°C, respectively. The measured values of T_g_ and T_m_ for electrospun TE-free fibers are higher than those of PLA, which has not been subjected to electrospinning. This is consistent with published data from Tsuji et al. (24). Obviously, dissolving PLA in a mixture of DCM and DMSO and subsequent evaporation of the solvent within an electrical field provokes a somewhat higher degree of order within the polymer. T_g_ and T_m_ values of TE-loaded fibers are slightly lower than those of TE-free fibers, which can be attributed to the intermolecular interaction between PLA and TE, indicating that TE acts as a kind of plasticizer for PLA. The same has been observed in electrospun drug-loaded PLA fibers (43) as well as Eudragit L100 fibers (36). The exothermic peaks of TE-free and TE-loaded fibers appearing near 121°C and 132°C separately belong to T_c_ of PLA, indicating the incomplete crystallization during the high-speed of electrospinning, while no such obvious peak is observed in the DSC curve of as-received PLA (44).

**Table 1:** Glass Transition Temperature (T_g_), Cold Crystallization Temperature (T_c_) and Melting Point (T_m_) of PLA, TE-free, and TE-loaded PLA Electrospun Fibers

	The first heating process			The second heating process		
	T_g_ (°C)	T_c_ (°C)	T_m_ (°C)	T_g_ (°C)	T_c_ (°C)	T_m_ (°C)
PLA	72		196	52.2		173.0
TE-free fiber	66	81	179, 186	60.6	121	177.3
TE-loaded fiber	68	83	177	60.1	132	171.3

**Fig. 5:**
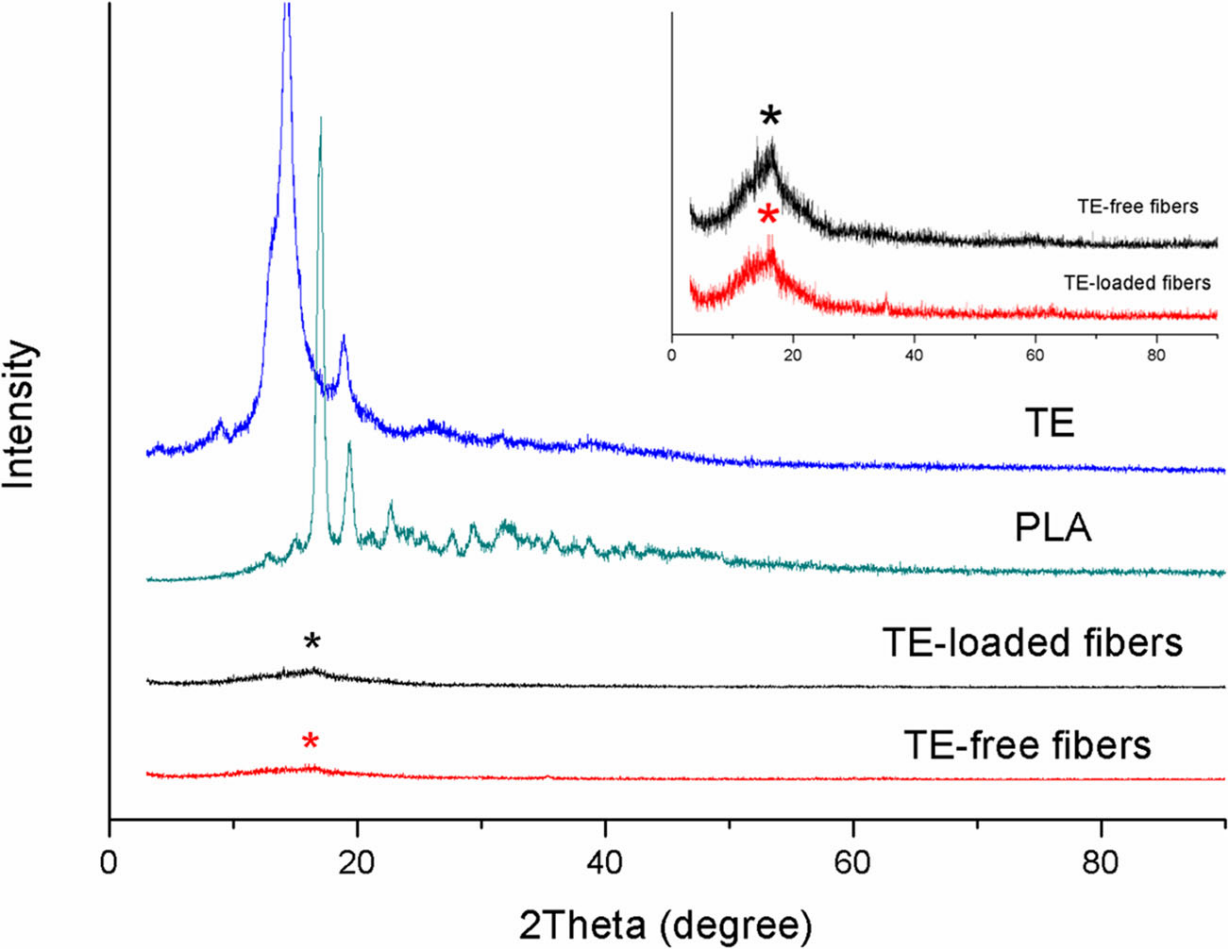
XRD patterns of TE, PLA, TE-free, and TE-loaded electrospun PLA fibers. This symbol (*) represents PLA peaks. The inset shows the PLA peaks of TE-free and TE-loaded fibers

Diffraction pattern of TE, PLA, TE-free, and TE-loaded electrospun PLA fibers is shown in Fig. 5. The main peaks of TE at 2θ = 12.86°, 14.36°, and 18.96° are approaching to those of betulin, especially at 2θ = 12.37°, 14.31°, and 18.66° (37,45). In the pattern of TE-loaded electrospun PLA fibers, no peak of TE can be found. It is considered due to the low ratio of TE to PLA, the decreased crystallinity of TE, and the change of TE into amorphous state as described in a report of curcumin-loaded electrospun fibers (47). For the pristine PLA, peaks present at diffraction angles of 2θ = 17.08° and 19.31°, revealing the presence of crystalline phases, whereas TE-free and TE-loaded electrospun PLA fibers display very low intensity peaks at diffraction angles of 2θ = 16.57° and 16.47°, respectively, indicating a fewer degree in crystallinity or that the crystallite size is too small to be detected by XRD (47–49).

### Dissolution Testing

**Fig. 6:**
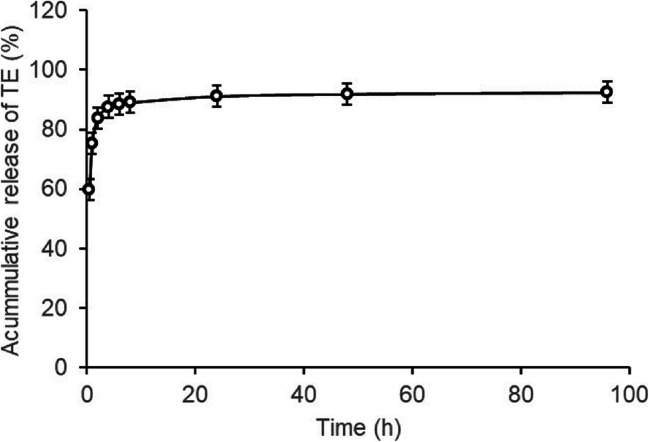
Accumulative release of TE from TE-loaded PLA electrospun fibers

The dissolution profile of TE-loaded PLA electrospinning fibers is given in Fig. 6. A substantial burst release can be observed at first followed by sustained release of the TE. The released profile is in good agreement with the results published for many drug-loaded electrospun fibers (50–52). More than 60% of TE released at 0.5 h. This burst release is within the range described in literature. It is higher than that of ciprofloxacin from electrospun fiber (50) but lower than that of itraconazole from electrospun fiber (53). In general, there are three major mechanisms of drug release from electrospun nanofiber PLA membranes: desorption, diffusion, and degradation (6). The biphasic manner of TE release is considered due to the initial desorption of drug from the outmost surface of fibers and subsequent diffusion-mediated release. The initial burst release of drug from electrospun fibers is reported to be desired for wound healing (51,52). The initial burst release of curcumin-loaded electrospun fibers is supposed to help in controlling early infection post orthopedic or shin surgery due to the effect of curcumin against biofilm of some bacteria (51). As TE also has an inhibitory effect on bacterial biofilms (54), the initial burst release of TE-loaded electrospun PLA fibers is thus potential to address the problem of early infection in wound healing. Additionally, the TE-loaded PLA fibers are hardly supposed to degrade within the period of dissolution tests referring to the report where *in vitro* degradation test was performed to a comparable PLA mesh (55), and the release mechanism of TE is considered not to be due to the degradation of PLA.

The most frequently used medium in dissolution tests of electrospun fibers is phosphate buffer solution (PBS) and sometimes organic solvent was added to PBS to facilitate the dissolution of drug when it is not readily to dissolve in water (56). In this study, ethanol was added to PBS to promote the release of TE. At 96 h, less than 8% of TE is not released from the PLA fibers, which is considered to be encapsulated deep inside the PLA fibers (50).

## CONCLUSION

TE-loaded PLA fibers were successfully prepared by electrospinning technology. TE was confirmed to be entrapped in the PLA electrospun fibers by CLSM. Consequently, dissolution test showed a sustained release, however, with a marked burst release. DSC and XRD data revealed that TE was either dispersed in PLA as amorphous solid or in a molecularly dispersed state. More than 90% of TE was released *in vitro* from the electrospun fibers with an initial burst release. The TE-loaded electrospun PLA fibers in this study show promising potential for use as wound dressing.
